# Determinants of susceptibility and tolerance of *Streptococcus pneumoniae* to hypothiocyanous acid

**DOI:** 10.1128/iai.00070-26

**Published:** 2026-03-16

**Authors:** Balázs Rada, Jorge E. Vidal

**Affiliations:** 1 Department of Infectious Diseases, College of Veterinary Medicine, The University of Georgia 70734, Athens, Georgia, USA; 2 Department of Cell and Molecular Biology and Center for Immunology and Microbial Research, School of Medicine, The University of Mississippi Medical Center 21693, Jackson, Mississippi, USA; University of California Davis, Davis, California, USA

**Keywords:** HOSCN, *Streptococcus pneumoniae*, hypothiocyanite, microbicidal, pneumococcus

## Abstract

Infections caused by *Streptococcus pneumoniae* (*Spn*, pneumococcus), manifesting as pneumonia, meningitis, and otitis media, represent a leading cause of severe morbidity and mortality globally. Escalating antibiotic resistance and serotype replacement by conjugate vaccines underscore the need for serotype-independent therapies. Hypothiocyanous acid (HOSCN), a reactive oxidant naturally produced by the host innate immune system, is formed through the interaction of hydrogen peroxide, thiocyanate anions (SCN^−^), and peroxidase enzymes. HOSCN is produced at mucosal surfaces, including the respiratory epithelium, where it functions as a broad-spectrum oxidative agent. HOSCN has been reported to kill Spn *in vitro*, but tolerance mechanisms of Spn against HOSCN have also been described. This review synthesizes recent advances in understanding HOSCN’s molecular mechanisms of action, tolerance pathways of Spn, and the effects of HOSCN on host-Spn interactions. The article also discusses current limitations and key challenges that must be addressed to translate HOSCN from preclinical promise to clinical application in the management of Spn lung infections. By leveraging an innate microbicidal pathway already active in the healthy lung, HOSCN-based therapeutics could offer rapid, resistance-refractory control of pneumococcal disease.

## INTRODUCTION

*Spn* is a gram-positive, lancet-shaped diplococcus (spherical bacterium typically appearing in pairs) (1, 2). The most critical virulence factor of *Spn* is its polysaccharide capsule that surrounds the cell and acts as a major antiphagocytic barrier, allowing the bacterium to evade the host immune system (1, 3). There are over 100 known capsular serotypes (2, 4, 5). *Spn* is a facultative anaerobe microorganism exhibiting alpha-hemolysis on blood agar plates by oxidizing oxy-hemoglobin to met-hemoglobin (6), and it is catalase negative, distinguishing it from *Staphylococcus* species (1). A key laboratory test is its sensitivity to the antibiotic optochin and its solubility in bile, which helps differentiate it from other *Streptococcus* species (1). Beyond its capsule, other *Spn* virulence factors include pneumolysin (a cytotoxin that damages host cells and activates inflammation), adhesins (proteins that help the bacteria attach to respiratory epithelial cells), and IgA protease (degrades the host’s IgA antibodies on mucosal surfaces) (1, 2).

*Spn* is an opportunistic pathogen colonizing mucosal surfaces in the human upper respiratory tract. Up to 65% of children and 10% of adults are *Spn* carriers (2, 7–9). While a commensal relationship exists between *Spn* and the host under normal conditions, spread of *Spn* into other sites, including the lung, middle ear, blood, and meninges, leads to invasive inflammatory diseases (2, 7, 8). *Spn* is a leading bacterial cause of a broad range of infections, including community-acquired pneumonia, meningitis, otitis media, and sepsis (2, 10). Although the widespread use of pneumococcal conjugate vaccines has reduced invasive disease of serotypes with the capsular polysaccharide types included in the vaccine (2, 11), the genetic adaptability of *Spn* has facilitated the spread of antibiotic resistance and evasion of vaccine-induced immunity (2, 10, 12).

Pneumococcal vaccines have been used since the 1980s (2). The most recently licensed pneumococcal vaccines include PCV20 (Prevnar 20), approved for both children and adults, which provides coverage against 20 different serotypes (1, 3, 4, 5, 6A, 6B, 7F, 8, 9V, 10A, 11A, 12F, 14, 15B, 18C, 19A, 19F, 22F, 23F, and 33F) and PCV21 (CAPVAXIVE), which is licensed for adults in the USA and targets additional serotypes not included in PCV20 (15C, 16F, 17F, 20, 23A, 23B, 24F, 31, and 35B) (2, 13–15). The use of pneumococcal conjugate vaccines has led to a decrease in the cases of severe pneumococcal pneumonia in many countries (2). However, infections caused by non-vaccine types have concurrently increased, leading to severe pneumonia and hampering the effect of vaccinations (2, 16–18).

Antibiotic-resistant pneumococcal infections account for an estimated $233 million in total healthcare cost in the USA (19). *Spn* resistant to at least one antibiotic causes more than two in five *Spn* infections, and resistance to macrolide antibiotics represents the majority of the cases (CDC.gov, all ages, 1998–2011). Macrolide-resistant *Spn* is a top priority pathogen on the WHO’s list of pathogens resistant to antibiotics (2017 and 2024).

Thus, *Spn* remains to be a significant problem to society due to its high clinical relevance and emerging resistance to antibiotics (2).

### Hypothiocyanous acid

**Fig 1:**
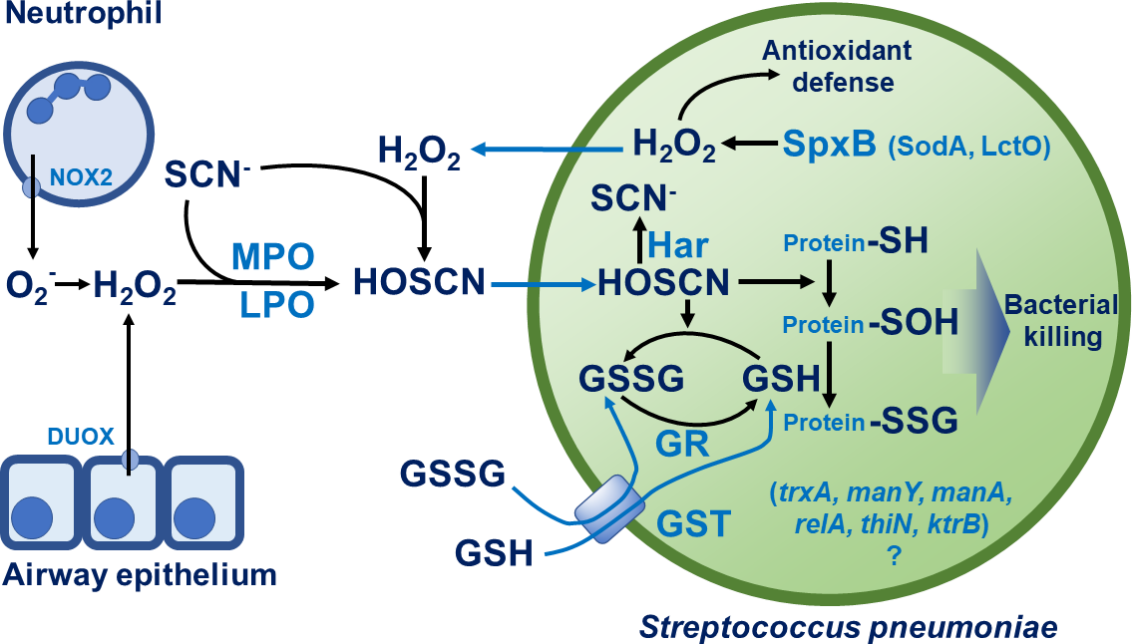
HOSCN production and *Streptococcus pneumoniae* defense mechanisms in the airway. HOSCN is produced in the airways by lactoperoxidase (LPO) oxidizing SCN^−^ with DUOX-derived H_2_O_2_ generated by airway epithelial cells or by neutrophil-derived myeloperoxidase (MPO) that oxidizes SCN^−^ with NOX2-derived H_2_O_2_ (produced via O_2_^−^). HOSCN kills pneumococcus likely by oxidizing bacterial protein thiols (^−^SH → ^−^SOH). *S. pneumoniae* resists the attack by HOSCN via (i) Har, an NADPH- and flavin-dependent HOSCN reductase; (ii) redundant glutathione (GSH) system (import, GST, GR) that repairs oxidized proteins (e.g., protein-SSG reduction); and (iii) endogenous H_2_O_2_ from pyruvate oxidase (*SpxB*, minor *LctO* contribution), which paradoxically increases tolerance to exogenous HOSCN (mechanism unclear). Additional putative tolerance genes include *trxA*, *manY/manA*, *relA*, *thiN*, and *ktrB*. Solid arrows, reactions/production; blue lines, molecular transport across bacterial cell wall and membrane; blue text, proteins; ?, unresolved role; GR, glutathione reductase, glutathione S-transferase.

Hypothiocyanous acid (HOSCN) is a potent antimicrobial agent produced by the body’s innate immune system at mucosal surfaces, found in fluids like the airway surface liquid, saliva, and milk. HOSCN has been reported to kill several pathogens *in vitro* (see our comprehensive review [20]). HOSCN is formed when peroxidases oxidize thiocyanate (SCN^−^) using H_2_O_2_. SCN^−^ is a ubiquitous pseudohalide derived from either the environment (food, enriched in cruciferous vegetables, buckwheat, cassava, sweet potatoes, and lima beans) or from metabolism in the host (21). SCN^−^ is present in the blood and highly enriched in mucosal secretions (Fig. 1), including the airway surface liquid (21, 22). Plasma SCN^−^ levels are 5–70 µM (23), while SCN^−^ levels in the saliva and airway surface liquid can reach millimolar values (21). The main peroxidase dominant in the airways under normal conditions is lactoperoxidase (LPO) (24, 25). LPO is released by the airway epithelium and submucosal glands (26, 27). LPO can oxidize several substrates using H_2_O_2_, but its main substrate is SCN^−^ to form HOSCN (Fig. 1) (25, 28). LPO does not use Cl^−^ as a substrate and does not form HOCl, unlike the dominant peroxidase found under inflammatory conditions in the airways, myeloperoxidase (MPO) (29). MPO is highly expressed in neutrophils (30). In pathological, inflammatory settings characterized by excessive neutrophil infiltration, MPO is released into the airway lumen, making it the dominant airway peroxidase, and its HOCl production is a significant contributor to lung tissue damage (31, 32). LPO, MPO, and SCN^−^ are all non-toxic host molecules and naturally present in human tissues (33).

The precise molecular mechanism by which HOSCN kills microbes remains poorly understood. It is suspected that HOSCN targets and oxidizes critical thiol groups in bacterial proteins, which form the molecular basis of its antibacterial action (34, 35). The precise molecular mechanism of HOSCN likely varies among species and mainly depends on the oxidative targeting of proteins that are crucial for the survival of the certain pathogen. Our latest review article has provided an updated, detailed summary of the literature reporting molecular targets of HOSCN in microbes, including *Spn* (36).

## EVIDENCE OF SUSCEPTIBILITY OF SPN TO HOSCN

We were the first to demonstrate that *Spn* is susceptible to HOSCN (37). The study by Gingerich et al. showed that LPO-generated HOSCN had both bactericidal and bacteriostatic effects on encapsulated and non-encapsulated pneumococcal strains *in vitro* (37). Catalase, an H_2_O_2_ scavenger, inhibited pneumococcal killing by HOSCN under all circumstances, indicating the essential role of H_2_O_2_ in the process (37). Interestingly, the presence of the bacterial capsule or LytA-dependent autolysis had no effects on HOSCN-mediated *Spn* killing (37). Overall, numerous pneumococcal strains, including vaccine-escape strains and multidrug-resistant strains, are susceptible to HOSCN *in vitro*.

## MECHANISMS OF TOLERANCE OF SPN TO HOSCN

While the work above reported *in vitro* evidence of *Spn* susceptibility to HOSCN, other studies indicated that *Spn* has some tolerance toward HOSCN, especially when compared to other respiratory pathogens like *Pseudomonas aeruginosa* (38, 39).

### Hypothiocyanous acid reductase

Shearer et al. documented the enzymatic reduction of HOSCN by *Spn* lysates, which only took place in the presence of NADPH, was independent of glutathione (GSH) reductase or thioredoxin reductase (Fig. 1), and was tightly linked to flavin adenine dinucleotide (39). The deletion of the *SPD_1415*/*har* gene encoding a putative flavoprotein disulfide reductase abolished HOSCN reductase activity in *Spn* (39). Thus, the authors identified a novel gene and enzyme, *har*/hypothiocyanous acid reductase (Har), that specifically targets and degrades HOSCN *in vitro* in *Spn* (39).

### Synergy with the GSH system

Har-associated HOSCN reductase activity was, however, dispensable for *Spn* growth, in the presence of LPO and SCN^−^ (39). Interestingly, *Spn* growth in the HOSCN-generating system was only completely blocked when *har* deletion was combined with the deletion of GSH transferase (39). *Har* deletion, in combination with the disruption of GSH import and recycling, significantly sensitized *Spn* to HOSCN (Fig. 1). These results demonstrated a role for Har, in combination with GSH utilization, to protect *Spn* from HOSCN and indicate that Har and GSH each provide alternate, redundant mechanisms to target HOSCN.

### Pyruvate oxidase (SpxB)

In the oxygen-rich environment of the lungs, *Spn* relies on fermentative metabolism, producing ATP by oxidizing glucose to acetate (40). *Spn* strains possess an incomplete set of genes for the Krebs cycle and respiratory chain, with ATP production partly driven by pyruvate oxidase (SpxB), which generates H_2_O_2_ as a byproduct (41). We have reported that *Spn* mutants deficient in *spxB* demonstrated reduced H_2_O_2_ production (~20% of wild-type strain) and displayed enhanced susceptibility to HOSCN, compared to its parental strain (Fig. 1) (37). *Spn*-H_2_O_2_ has also been implicated in cytotoxicity to lung cells, induction of apoptosis, and other toxic events observed during bacterial invasion of the central nervous system and heart, although the precise mechanisms remain to be fully elucidated (42, 43). Mutants lacking *spxB* produce less capsule due to the absence of acetylated capsule precursors and show attenuated virulence in mouse models of pneumococcal disease (41, 44). The majority of *Spn* strains in which deficiency in *spxB* conferred a capsule defect were of serotype 4, but capsule production was not affected when other important types were assessed, including vaccine type 19F. Encapsulated *spxB* mutants were attenuated for virulence (41, 44, 45). For example, a D39-derivative *spxB* mutant was attenuated in both pneumonia and bacteremia models in rabbits (45), and in Balb/c mice, mutants without capsule defects exhibited delayed mortality and significantly lower bacterial counts in blood (41).

*Spn*-H_2_O_2_ is a potent oxidant; in co-culture experiments, *Spn* rapidly killed *Staphylococcus aureus* or *Haemophilus influenzae* (46, 47). This bactericidal effect is primarily attributed to SpxB, though LctO contributes to H_2_O_2_ production under certain conditions, as Δ*spxB* mutants can still kill *S. aureus* (48). We recently demonstrated that H_2_O_2_ generated by LctO in the absence of SpxB produces hydroxyl radicals (^•^OH) sufficient to kill *S. aureus*. This effect was abolished in double Δ*spxB*Δ*lctO* mutants or when ^•^OH was scavenged with thiourea or salicylate (49). There is substantial evidence that SpxB drives oxidative stress *in vivo*. The alveolar-capillary network provides erythrocytes and hemoglobin, which have been shown to enhance *Spn* growth *in vitro* and upregulate *spxB* expression. SpxB-derived H_2_O_2_ oxidizes oxy-hemoglobin (Fe²^+^) to met-hemoglobin (Hb-Fe³^+^), generating toxic species, such as labile heme and hemoglobin-derived tyrosyl radicals (^•^Hb-Fe⁴^+^) (6, 50). Notably, mutants deficient in H_2_O_2_ production were highly susceptible to physiological levels of heme-mediated toxicity (50). Overall, these data indicate that SpxB plays relevant roles in *Spn* survival, including enhanced tolerance to HOSCN-mediated oxidative damage, but the details of SpxB-mediated increased tolerance remain entirely unknown.

### Additional genes associated with HOSCN tolerance

Shearer et al. used saturation transposon mutagenesis and deep sequencing to find genes of Spn tolerance to HOSCN (51). Thirty-seven genes were identified that were associated with *Spn* HOSCN tolerance, including genes involved in metabolism, membrane transport, DNA repair, and oxidant detoxification (51). The activities of Har or glutathione reductase, known to be important for *Spn* tolerance of HOSCN, were enhanced in three of the mutants, highlighting the compensatory potential of antioxidant systems (51). The HOSCN defense systems identified in this study could be exploited to improve the sensitivity of *Spn* to HOSCN.

## *IN VIVO* STUDIES

Most reports focusing on the interaction between *Spn* and HOSCN were performed *in vitro*. It remains largely unknown whether HOSCN is effective against *Spn* lung infection *in vivo* and whether the identified *in vitro* resistance mechanisms also provide an advantage to the bacterium in a mammalian host. A recent study performed by Shearer et al. aimed at filling in this gap and studied the effects of *har* and *gshT* in the *in vivo* virulence of *Spn* (52). The report demonstrated in mouse models of *Spn* lung infection that *har* was essential for colonization and invasion (52). In a colonization model, *Spn* titers in the nasopharynx dropped drastically when *har* was deleted in *Spn* (52). The Δ*har* Spn strain was also less virulent, compared to the wild-type control, in an invasion model in the lungs, and no dissemination to the blood or brain was observed (52). These results indicate that pneumococcal Har is an efficient HOSCN reductase, increasing bacterial survival in the presence of HOSCN *in vivo*.

## THERAPEUTIC IMPLICATIONS

This review summarizes current knowledge in the field of HOSCN-*Spn* interactions and indicates that HOSCN represents a potent, non-antibiotic mechanism targeting bacteria including *Spn*. Studying HOSCN is also relevant due to its potential to combat antimicrobial resistance. HOSCN acts through a mechanism independent of currently approved antimicrobials, positioning it and its precursor SCN^−^ as a promising therapeutic avenue against antibiotic-resistant strains of *Spn*, a pathogen listed by the WHO as a global priority. HOSCN is better tolerated by host tissue than other, more aggressive oxidants like hypochlorous acid, potentially offering a higher therapeutic index and higher chance for safe *in vivo* applications. Despite the potent antimicrobial nature of HOSCN, *Spn* exhibits some tolerance to HOSCN, compared to other respiratory pathogens. Bacterial tolerance to HOSCN could be diminished, for instance, by inhibiting *Spn* proteins that have been associated with HOSCN resistance. Potential targets include Har and the main hits of the genome-wide screen that identified several genes associated with HOSCN tolerance in *Spn*. Therefore, further studies are needed to explore whether HOSCN can improve the clinical outcomes of pneumococcal pneumonia and is capable of fighting antimicrobial resistance.
